# Anthropogenic Habitats Facilitate Dispersal of an Early Successional Obligate: Implications for Restoration of an Endangered Ecosystem

**DOI:** 10.1371/journal.pone.0148842

**Published:** 2016-03-08

**Authors:** Katrina E. Amaral, Michael Palace, Kathleen M. O’Brien, Lindsey E. Fenderson, Adrienne I. Kovach

**Affiliations:** 1 Department of Natural Resources and the Environment, University of New Hampshire, Durham, New Hampshire, United States of America; 2 Institute for the Study of Earth, Oceans, and Space, University of New Hampshire, Durham, New Hampshire, United States of America; 3 United States Fish and Wildlife Service, Rachel Carson National Wildlife Refuge, Wells, Maine, United States of America; 4 United States Fish and Wildlife Service, Northeast Fishery Center, Conservation Genetics Lab, Lamar, Pennsylvania, United States of America; Clemson University, UNITED STATES

## Abstract

Landscape modification and habitat fragmentation disrupt the connectivity of natural landscapes, with major consequences for biodiversity. Species that require patchily distributed habitats, such as those that specialize on early successional ecosystems, must disperse through a landscape matrix with unsuitable habitat types. We evaluated landscape effects on dispersal of an early successional obligate, the New England cottontail (*Sylvilagus transitionalis*). Using a landscape genetics approach, we identified barriers and facilitators of gene flow and connectivity corridors for a population of cottontails in the northeastern United States. We modeled dispersal in relation to landscape structure and composition and tested hypotheses about the influence of habitat fragmentation on gene flow. Anthropogenic and natural shrubland habitats facilitated gene flow, while the remainder of the matrix, particularly development and forest, impeded gene flow. The relative influence of matrix habitats differed between study areas in relation to a fragmentation gradient. Barrier features had higher explanatory power in the more fragmented site, while facilitating features were important in the less fragmented site. Landscape models that included a simultaneous barrier and facilitating effect of roads had higher explanatory power than models that considered either effect separately, supporting the hypothesis that roads act as both barriers and facilitators at all spatial scales. The inclusion of LiDAR-identified shrubland habitat improved the fit of our facilitator models. Corridor analyses using circuit and least cost path approaches revealed the importance of anthropogenic, linear features for restoring connectivity between the study areas. In fragmented landscapes, human-modified habitats may enhance functional connectivity by providing suitable dispersal conduits for early successional specialists.

## Introduction

Landscape connectivity is vital for species persistence, as it facilitates the movement of individuals and their genes and facilitates ecological processes and resources through the landscape [1]. Landscape modification and habitat fragmentation disrupt the structural connectivity of natural landscapes, with major consequences for biodiversity [2,3]. Connectivity issues are germane to species living in naturally patchy and ephemeral habitats [4,5]. The spatial configuration of patchily distributed habitat poses connectivity challenges, and species dependent on these habitat types are likely to respond to landscape features differently than generalist species [6,7].

Landscapes consisting of early successional (shrubland) habitats are ideal for investigating fragmentation effects on animal dispersal. These ephemeral habitats are patchy by nature and occur in a heterogeneous landscape matrix comprised of a diversity of habitats, many of which are inhospitable to early successional specialists. Due to a loss of natural disturbance regimes, land use change, and anthropogenic landscape modifications, early successional habitats are on the decline in eastern North America [8–11]. Species reliant on these declining habitats face consequences of habitat loss and fragmentation, including population isolation and decline [12–15]. As such, early successional ecosystems are among the most endangered, and their conservation is a high priority in North America [11] and elsewhere [16].

In these fragmented early successional systems, where shrubland habitat is limited, functional connectivity may be maintained by anthropogenic habitats that provide suitable dispersal conduits. For example, areas in which periodic human activity such as mowing or cutting occurs may hinder forest succession and provide consistent early successional habitat. These human-modified habitats often occur in narrow, linear strips, such as along roadsides or utility lines, and may provide movement corridors for shrubland species [4,17,18], much like riparian corridors can provide dispersal pathways for aquatic or forest specialist species [19–20]. Roads and other linear landscape features, typically thought to be barriers to animal movement [21–22], may therefore facilitate movement in some species or act as both dispersal barriers and facilitators within a single species [2].

The matrix surrounding early successional habitat patches may also contain natural habitat types or landscape features that, while not optimal for species’ occupancy, may enhance connectivity by providing stepping-stone patches for dispersal. Such features may include wetlands with herbaceous cover, grasslands, agricultural lands, and old fields. Whether these less densely vegetated habitat types provide suitable cover to facilitate connectivity of early successional habitat specialists remains unknown and likely varies with the degree of the organism’s habitat specialization.

To address issues of connectivity in early successional ecosystems, we investigated landscape effects on dispersal of an obligate species of high conservation concern, the New England cottontail (*Sylvilagus transitionalis*). Along with many other shrubland specialists, the New England cottontail has experienced recent population declines, mirroring range-wide losses in habitat [23]. It is a species of greatest conservation need in every state in which it occurs, listed as endangered in the states of Maine and New Hampshire [24,25], and was a candidate for federal listing under the Endangered Species Act until a recent decision determined that current conservation efforts were sufficient to forego listing [26]. These conservation efforts rely on extensive habitat creation and restoration. Yet the empirically based knowledge of cottontail dispersal necessary to guide the design of restoration landscapes is minimal. To this end, our primary goals were to develop testable hypotheses about the functional connectivity of an early successional obligate and to generate knowledge to guide restoration activities for this threatened species and ecosystem.

We evaluated the landscape matrix features, including both anthropogenic and natural habitats, in relation to New England cottontail dispersal and in the context of landscape heterogeneity and fragmentation. We used a landscape genetics approach to model observed gene flow (effective dispersal) in relation to landscape structure. Based on previous research as well as expert opinion, we hypothesized that major roads, development, water, open fields, and mature forests would act as barriers to dispersal, while wetlands, scrub-shrub landcover, and linear, anthropogenic, shrubby habitat features such as powerlines, railroad crossings, and roadsides, would facilitate dispersal (Table 1). To evaluate the dual influence of roads as both barriers and facilitators, we developed a model that simultaneously accounted for these opposing effects on dispersal. We also included a model with LiDAR-detected habitat and predicted that it would improve facilitator model fits. We evaluated landscape heterogeneity and fragmentation effects by comparing two metapopulations occupying landscapes with different compositions and configurations. We hypothesized that barrier landscape features would predict gene flow in the more fragmented landscape and facilitating features would be more influential in the less fragmented landscape. Lastly, we used the results of our landscape genetics analyses to identify potential movement corridors and key areas for restoration within and between the two study areas. Specifically, we were interested in the potential for anthropogenic habitats to enhance functional connectivity via human-modified, linear, dispersal corridors.

**Table 1:** Landscape influences on cottontail gene flow. Landscape features evaluated in this study along with their hypothesized and empirically identified (from univariate least cost path models) influence on New England cottontail gene flow. Plus signs indicate postive relationship, minus signs indicate negative relationship.

Landscape Variable	Hypothesized Relationship to Gene Flow	Identified Relationship to Gene Flow
Roads^1^	**-**	**-**
Development	**-**	**-**
Fields	**-/+**	**-**
Forest	**-**	**-**
Water	**-**	**-**
Scrub/Shrub	**+**	**+**
Forested Wetlands	**+**	**-**
Scrub/Shrub Wetlands	**+**	**+**
Estuarine Emergent Wetlands	**+**	**-**
Palustrine Emergent Wetlands	**+**	**+**
Linear Facilitators^2^	**+**	**+**
LiDAR-detected habitat^3^	**+**	**+**

^1^Road crossings

^2^Powerlines, railroad corridors and roadsides

^3^LiDAR-detected habitat data were only available for Cape Elizabeth

## Methods

### Study system

The New England cottontail, an early-successional-habitat obligate, is a model organism for studying connectivity in heterogeneous landscapes and investigating fragmentation effects on dispersal. Once widespread throughout the New England states and eastern New York, New England cottontails today are found in five geographically isolated and genetically distinct populations located in southern Maine and southeastern New Hampshire, central New Hampshire, eastern Massachusetts on Cape Cod, eastern Connecticut and Rhode Island, and western Connecticut and New York [23,27]. Remnant populations of New England cottontails today occupy less than 14% of their historical range and less than 10% of the remaining habitats within this range [23]. These remaining habitats are small and discontinuous and exist as discrete patches within gradients of fragmentation resulting from ongoing anthropogenic landscape modifications. Within this landscape, New England cottontails function as metapopulations, with their persistence dependent on connections among networks of suitable habitat patches [28].

In this study, we focus on a geographically isolated group of cottontails at the northern extent of the species range in southern Maine and New Hampshire. This study area has been the subject of recent occupancy [29] and population genetics [30] research. Cottontails in this region have experienced range contraction and population bottlenecks, and they have reduced genetic diversity relative to other cottontail populations [27,30]. They occur in a landscape in which remaining habitat patches are small (2–35 ha, mean = 5 ha) and fragmented by development and inhospitable habitat. In previous research, major highways and large waterbodies were found to impede dispersal and isolate metapopulations, while the shrubby habitat along roadsides, railroad beds, and utility corridors to facilitate cottontail dispersal within populations [30]. The importance of these linear dispersal barriers and facilitators relative to the other features of the landscape matrix, however, is unknown, as are the principal factors that influence gene flow in this system. Here we investigate the full suite of landscape features (Table 1) in relation to gene flow in a fragmentation gradient.

**Fig 1:**
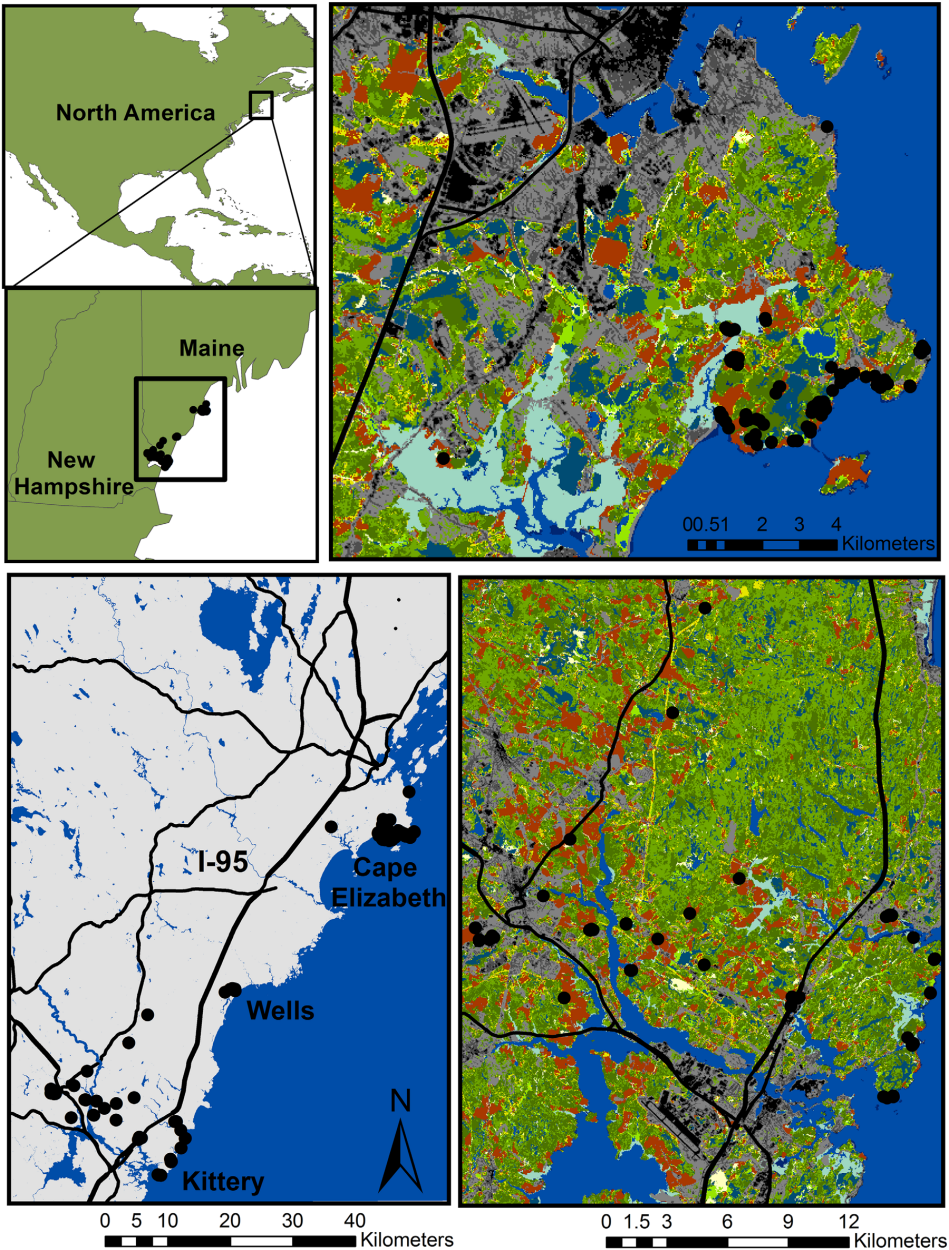
Study area in Maine/New Hampshire (USA). Top left two insets provide context for study area location in North America within the states of Maine and New Hampshire. Bottom left panel shows the full extent of the study area. I-95 is shown by solid black line partitioning east and west sides of the Kittery region. The Piscataqua River is visible in the southern portion of Kittery. Close ups of the two study study areas with landcover are shown in the top right for Cape Elizabeth and bottom right for Kittery. Locations of sampled New England cottontail individuals are shown by black points. Landcover key: gray = development, green = forest, orange = fields, yellow = scrub/shrub, dark blue = open water, and light blue = wetlands.

Within this landscape, cottontails occupy remnant patches primarily in two geographically distinct areas, with no current gene flow between them, although historically there were occupied intervening patches [23,30]. A northern metapopulation in Cape Elizabeth, southeast of the city of Portland, Maine, consists of a network of relatively close habitat patches, within a spatial extent of 8 by 13 km. It is comprised of a heterogeneous matrix of landcover types dominated by forest and suburban development but has no major highways or other high traffic volume roads. The second occupied area (hereafter "Kittery") is 40 km south, encompassing the towns of Kittery, York, and the Berwicks in Maine and Dover, New Hampshire, with a spatial extent of 18 by 23 km in a predominantly rural/agricultural landscape. Interstate 95 (I-95) and the Piscataqua/Salmon Falls River further subdivide this area. Cottontail densities are lower in Kittery, and habitat patches are, on average, smaller and more widely dispersed than those in Cape Elizabeth. Scrub-shrub, the preferred cottontail habitat, comprises 3.9–4.6% of each landscape, while dominant landcover features differ, with more development and wetlands in Cape Elizabeth and more forest and roads in Kittery (Table 2). Another small, isolated group of cottontails occurs on a few patches in Wells, Maine, located roughly halfway between the two populations (Fig 1).

**Table 2:** Study area composition and configuration. Characteristics of occupied patches and proportion of each study area comprised by specific landcover types. Road and LiDAR percentages indicate the overall proportion of landscape that they cover and their coverage overlaps with that of other landcover types.

Landcover	Kittery	Cape Elizabeth	Full study area
Development	11.2%	28.6%	14.7%
Fields	11.1%	8.9%	12.0%
Forest	58.8%	33.9%	54.3%
Scrub/Shrub	4.6%	3.9%	4.7%
Forested Wetlands	7.4%	9.3%	8.0%
Scrub/Shrub Wetlands	1.5%	1.1%	1.5%
Palustrine Emergent Wetlands	1.0%	2.1%	1.1%
Estuarine Emergent Wetlands	1.0%	9.2%	1.7%
Water	3.3%	3.1%	2.1%
Roads	25.3%	14.3%	24.9%
**Patch Characteristics**			
Patch Size (ha)	3.10	5.40	---
Perimeter:Area Ratio (edge)	557	414	---
Nearest Neighbor Distance (km)	1.45	0.33	---

### Ethics Statement

No vertebrate subjects were used in this research, as all genetic samples were obtained from fecal pellets that were noninvasively collected in the wild. All samples were collected with appropriate permits: the United Sates Fish and Wildlife Service authorized permission for collections on federal lands, Maine Division of Fisheries and Wildlife and New Hampshire Fish and Game authorized permission for collections on state lands, and landowners gave permission for collections on private lands. The focal organism of this study, the New England cottontail is a state endangered species in New Hampshire and Maine and was a candidate for federal listing under the Endangered Species Act at the time of this study. The United States Fish and Wildlife Service recently made the decision not to list the species.

### Data Availability

Microsatellite genotype and sample location data are available in the DRYAD data repository (http://dx.doi.org/10.5061/dryad.1s834). Pair-wise individual genetic distance data (Rousset’s *a* and *Dps*) are provided in S1 Appendix. Results of supporting data analyses (e.g., univariate resistance modeling optimization, Mantel’s tests results) are provided in S1–S4 Tables.

### Sampling and genetic data

For this study, we used a previously published dataset, consisting of genotypes at 11 microsatellite loci of 137 individuals sampled from unique georeferenced locations during intensive, systematic, fecal pellet surveys of occupied patches in 2007–2009 (see [30] for details of sampling and genotyping and Fig 1 for sampling locations). For identifying and comparing landscape influences on gene flow, we focused separately on the two primary geographic areas (Cape Elizabeth, n = 84, and Kittery, n = 48 –excluding the 5 individuals in Wells). To identify potential movement corridors for restoring connectivity among populations, we used all individuals (n = 137 total). To estimate gene flow among cottontails, we used two individual pairwise genetic distance metrics—Rousset's *a* [31] and *Dps* [32], which we calculated for all pairs of cottontails within each geographic area, separately. Rousset's *a* was calculated in Spagedi v1.4 [33] and *Dps* was calculated in Microsatellite Analyzer (MSA 4.05; [34]; pair-wise *a* and *Dps* values are in S1 Appendix). Euclidean distances between all pairs of sampling locations were calculated in R [35]. All subsequent analyses were conducted with the individual cottontail sampling location and individual cottontail genotype as the units of analyses.

### Overview of Landscape Genetics Approach

To evaluate landscape influences on gene flow, we used a resistance surface approach with a cost surface parameterized by the genetic data to model the movement of individuals between locations [7,36]. To model resistance to movement within each of the two study areas, we used least cost path distances [37] as our ecological distances between locations. Least cost paths assume that animals move in single paths that minimize resistance through the landscape. We followed a 2-step process, whereby we first developed univariate models, with binary resistance surfaces, for the influence of each hypothesized landscape feature on gene flow. We optimized resistance surfaces empirically, following [38], with a constrained optimization approach to identify the optimal resistance value for each landscape feature on gene flow; this optimization was done for each of the two study areas separately, using the same array of resistance values (see below). We then developed a set of *a priori* multivariate models, using resistance surfaces built from the univariate optimization results, to compare combinations of features representing anthropogenic and natural barriers and facilitators. To evaluate these models, we used linear mixed effects models and an information-theoretic approach for model selection. Lastly, to identify connectivity corridors for focusing restoration, we used the resistance surface from the best-supported multivariate landscape model and applied it across the entire study area with corridor analyses using both a least cost path and a circuit theory approach.

### Landscape Resistance Surfaces

We developed resistance surfaces for ten landscape variables selected for their hypothesized ability to impede or facilitate dispersal (Table 1). In addition, roadsides, powerline rights of way, and railroads, which are comprised of shrubby habitat, were mapped individually as well as considered together as linear facilitating features. Landcover variables were derived from the NOAA C-CAP 2006 landcover map and mapped at 30 m resolution. Wetland types were identified from the National Wetland Inventory. Roads, powerlines, and railroads were selected from a 1986 transmission shapefile (USGS 1989), which contained standardized road information between Maine and New Hampshire. We modified the layer to reflect current road structure and traffic-volume-based classification using current Maine state road data (Maine Department of Transportation, 2011). We considered separately the influence of 6 road classes, distinguished by traffic volume, with road class 1 corresponding to multi-lane highways and road class 6 corresponding to unpaved and unmaintained roads and trails. All roads were considered to be 30-m wide to match the 30 m resolution of the landcover layer, and class 1 roads were buffered to 60 m to reflect their true size relative to minor roads.

We also evaluated the role of habitats identified by LiDAR imagery. LiDAR is capable of identifying vegetation structure at a higher resolution than Landsat imagery [39], and its capacity for detecting vegetation less than ten meters in height is ideal for identifying the vegetation used by cottontails. LiDAR point cloud data (ground points identified by vendor) were acquired from FEMA (Federal Emergency Management Agency) and were available for the Cape Elizabeth study area only. Raw data was processed using the program Fusion v. 2.70 [40] to develop a canopy model and ground filter model to generate a surface model. Subtracting the ground surface from the canopy model resulted in 1-meter grids of vegetation at 1–3 meter height. GIS layers (ArcGIS 9.3) were generated and post processed using a nearest neighbor approach.

### Univariate Resistance Modeling

Each landscape variable was first tested as a separate, univariate resistance surface to identify how each landscape feature influenced cottontail gene flow (i.e., whether as a barrier or facilitator) and to generate optimal resistance values for use in subsequent multivariate modeling. For each study area, landscape variables were mapped separately in a binary friction grid and assigned elevated or reduced resistance costs relative to the background [41] based on their hypothesized effect on cottontail movement. Forest, open water, development, and roads were tested as barrier landcover types, using resistance values of 2, 5, 10, 25, 50, 100, 250, 500, 750, and 1000 against a background surface value of 1. Scrub/shrub habitat, linear features (roadside edges, powerlines and railroads), and LiDAR-detected habitats were tested as facilitating landcover features, with a resistance value of 1 against a background value of 100. Fields and wetlands were tested as both barriers and facilitators. Least cost path analyses for each univariate model were run in ArcMap (v10; Environmental Science Research Institute, Redlands, USA) using the landscape genetics toolbox (v1.2.3; [42]).

To optimize univariate resistance surfaces, we used Mantel and partial Mantel tests (compensating for the effects of geographic distance) to calculate correlations between effective distance (cumulative cost distance from least cost paths; [37]) and individual pairwise genetic distance (Rousset's *a* and *Dps*) in the ecodist R package [43]. For each landscape variable, we identified the unimodal peak of support in partial Mantel *r* values or the value at which the correlations began to plateau [38]; this represented the best fitting resistance value to the genetic data. While the efficacy of Mantel tests has been questioned for aspects of landscape genetics analyses, such as assessing model fit [44–46], they are powerful and appropriate for comparing distance matrices [47] and accurately identify drivers of genetic differentiation [48,49]. Further, they continue to be widely used, are easily interpretable, and provide a straight-forward approach for parameterizing resistance surfaces [50,51]. For these reasons, and recognizing that there is currently no consensus on the best analytical methods in landscape genetics, we used the partial Mantel tests to optimize our univariate resistance surfaces, but we did not use them to assess model fit or the relative importance of variables. For the latter purposes, we conducted linear mixed effects modeling on the cost distance outputs with individual pair-wise genetic distance as the dependent variable, using the *lmer* function in the lme4 R package [52], following [53]. We conducted model selection with AIC using the AICcmodavg R package [54], to compare the relative importance of individual variables. We also included a null model of geographic distance effects on genetic distance (isolation by distance; IBD).

### Multivariate Resistance Modeling

We developed a set of multivariate models to identify which combinations of landscape variables were most influential in New England cottontail gene flow. Rather than considering all combinations of binary variables, we built select multivariate models that included the most biologically relevant variable combinations. The goal of these models was to test the relative importance of barriers and facilitators as well as natural and anthropogenic features within each of the two landscapes. One of the variables of greatest *a priori* interest in our study was roads, and results of our binary models confirmed previous findings of [30] that roads function as both barriers and facilitators of gene flow. For this reason, we developed an approach for evaluating the simultaneous barrier and facilitating influences of roads. We assigned the pixels comprising the width of the roads the optimal barrier resistance costs from the univariate models, and then we buffered the roads by 30 m to include a single pixel width buffer with a cost value of 1 on each side, thereby modeling the effect of a road as a perpendicular barrier with roadside right-of-ways as parallel facilitators. To distinguish whether all road classes had this dual influence on dispersal or just the largest roads, we ran the full multivariate model with only road classes likely to include maintained right-of-ways buffered with facilitators and then again with all roads buffered with facilitators.

For each model, multivariate resistance surfaces were developed using the optimized resistances from the univariate models. The relative support for each resistance surface was evaluated using mixed effects models and AIC, as above. We applied mixed effect models to the least cost path distance outputs of our multivariate models (following [55]) rather than building the mixed effect models using individual landscape features.

### Corridor identification

Landscape features that structure metapopulations at the local scale may differ from those at the population level [56,57]. To evaluate these scale-dependent patterns, as well as to identify movement corridors that may have connected the two populations in the recent past and provide targets for future restoration, we tested multivariate landscape models across the full study area. As optimal resistance values differed for certain landscape features between the Cape Elizabeth and Kittery populations, we evaluated a few different models to identify the best fitting resistance values across this larger spatial extent. Specifically, we evaluated full models with all landscape features using population-specific optimal resistance values, as well as the average and maximum resistance values between the two study areas for each feature. Models included roads buffered with facilitators. As multi-lane highways and unmaintained roads were not present in the Cape Elizabeth landscape, for those road classes we only tested resistance values that were optimized for Kittery. We ran least cost path analysis for each set of resistance values and, as before, evaluated the relationship of effective cost distances and individual genetic distances using partial Mantel tests. We then used the set of resistance values that optimized the partial Mantel correlation to run the same suite of multivariate models that we ran for the two populations separately. We evaluated these competing multivariate models using mixed effect models and AIC as before. We used the least cost path outcome of the best fitting mixed effect model to identify corridors linking the Cape Elizabeth and Kittery populations.

As least cost path analyses are limited to identifying a single best movement pathway, for corridor analysis we also used a circuit-theoretic approach that enables identifying multiple paths simultaneously [58]. We implemented circuit analysis in Circuitscape v4.0 [59] across the entire study area to identify all potential movement corridors and important connectivity areas. For this analysis, we used the top multivariate resistance model identified from the full population least cost path analysis. Circuit models can be run across nodes (individual sampling locations) or focal patches (collections of cells that are considered together as a single node; [60]). We ran analyses between all individual sampling locations (137 nodes) as well as between focal patches that represented the core area of each metapopulation (3 nodes, 1 each in Cape Elizabeth, Wells, and Kittery). Models were run in the "all-to-one" mode, which is ideal for identifying important connectivity areas while minimizing run-time and memory usage [60]. Areas of high movement probability identified by circuit analysis were compared to the corridor pathways resulting from the least cost path analysis.

## Results

### Univariate Resistance Modeling

Tests using Rousset's *a* and *Dps* provided similar correlations; results from only Rousset's *a* are reported here. Overall, optimized barrier resistance values ranged from 2–250 and were highest for forested wetlands, followed by development and roads (S1 Table). As expected, optimal resistance values for landscape features varied between the Cape Elizabeth and Kittery regions, however, all landscape features were found to consistently exert either barrier or facilitating effects in the two regions. In contrast to our predictions, not all wetlands had the same effect on gene flow: scrub-shrub and palustrine emergent wetlands exhibited a positive effect on gene flow, while forested and estuarine wetlands exhibited a negative effect. Fields were also found to have a negative effect on gene flow. All other landscape features were found to have the predicted effect on gene flow (Table 1).

All features were significantly correlated with gene flow, even when controlling for distance, and all but the linear facilitator model in Kittery had higher Mantel *r* correlations than geographic distance alone (S2 Table). Development, roads, forest, and forested wetlands had higher resistance values in Cape Elizabeth, while water, estuarine emergent wetlands, and fields had higher resistance values in Kittery. Major roads (classes 1–3) had higher resistance values than minor roads (classes 4–6). Some roadsides were also positively correlated with gene flow when they were considered as univariate facilitators. In Kittery, including road classes 1–3 as facilitators along with powerlines and railroads produced the highest partial Mantel *r* correlation, and in Cape Elizabeth, class 3 roadsides were also significant as linear facilitators (there were no road classes 1,2 and 6 in Cape Elizabeth). Within the Cape Elizabeth landscape, LiDAR-identified habitat was significantly correlated with gene flow (r = 0.1398, p-value = 0.0004). Mixed effect models identified buffered roads as the top predictor of gene flow in Kittery (simultaneous barrier and facilitator effect) and scrub/shrub wetlands as the top predictor of gene flow in Cape Elizabeth (facilitating effect; Table 3).

**Table 3:** Univariate landscape model results. Model selection results for univariate linear mixed effects models of the relationship of landscape features on individual genetic distance, measured by Rousset’s *a*, for New England cottontails in the Kittery and Cape Elizabeth study areas. AICc is the second order or sample size corrected Akaike information criterion, delta AICc is the difference in AICc of each competing model relative to the best model, and AICcWt is the probability that the model is the best fit.

Model	AICc	Δ AICc	AICcWt
***Kittery***			
buffered_roads^1^	55.82	0.00	1.00
water	84.77	28.94	0.00
emergent_wetlands	103.72	47.89	0.00
estuarine_wetlands	107.16	51.34	0.00
fields	108.24	52.42	0.00
roads_barrier	109.75	53.92	0.00
development	109.80	53.97	0.00
forested_wetlands	111.36	55.54	0.00
shrub_wetlands	115.50	59.68	0.00
scrub_shrub	120.33	64.50	0.00
forest	122.20	66.37	0.00
roads_facilitator	133.99	78.16	0.00
null	389.11	333.29	0.00
***Cape Elizabeth***			
shrub_wetlands	-2098.25	0.00	1.00
development	-2073.28	24.98	0.00
roads_facilitator	-2068.01	30.24	0.00
scrub_shrub	-2067.66	30.59	0.00
fields	-2066.04	32.21	0.00
forest	-2066.02	32.23	0.00
estuarine_wetlands	-2063.96	34.29	0.00
water	-2063.89	34.37	0.00
emergent_wetlands	-2063.10	35.15	0.00
roads_barrier	-2058.33	39.92	0.00
lidar^2^	-2054.31	43.94	0.00
forested_wetlands	-2039.07	59.18	0.00
buffered_roads^1^	-2032.03	66.22	0.00
null	-2002.73	95.53	0.00

^1^ roads modeled as simultaneous barrier and facilitator with road width as barrier and 30 m strip buffered on either

^2^ 1–3 m tall vegetation detected by lidar imagery; data only available for Cape Elizabeth study area

### Multivariate Resistance Modeling

Including LiDAR-classified short stature vegetation as a facilitating landcover type in Cape Elizabeth models increased both Mantel and partial Mantel correlations (S3 Table). Models that included roads buffered with facilitators—to account for the simultaneous barrier and facilitator effects—always outperformed analogous models that only considered roads as barriers. These full models with buffered roads had the highest Mantel correlation when only road classes 1–3 were buffered as facilitators in Kittery, and with all road classes buffered in Cape Elizabeth (only 3,4,5 road classes present). Mixed effects models identified the all-barriers model as the best predictor of gene flow in Kittery, with 94% AIC model weight, and the model with both anthropogenic and natural facilitators was the most explanatory in Cape Elizabeth, with 98% AIC model weight (Table 4).

**Table 4:** Multivariate landscape model results. Model selection results for multivariate linear mixed effects models of the relationship of landscape features on individual genetic distance, measured by Rousset’s *a*, for New England cottontails in the Kittery and Cape Elizabeth study areas. AICc is the second order or sample size corrected Akaike information criterion, delta AICc is the difference in AICc of each competing model relative to the best model, and AICcWt is the probability that the model is the best fit.

	AICc	Delta_AICc	AICcWt
***Kittery***			
all_barriers^1^	87.92	0.00	0.94
landcover	93.66	5.74	0.05
global^2^	98.40	10.48	0.00
development + roads_barrier	111.26	23.34	0.00
natural_barriers^3^	120.07	32.16	0.00
natural_facilitators^4^	149.10	61.18	0.00
all_facilitators^5^	181.71	93.80	0.00
null	389.11	301.19	0.00
linear (anthropogenic) facilitators^6^	399.04	305.19	0.00
***Cape Elizabeth***			
all_facilitators	-2086.67	0.00	0.98
natural_facilitators	-2077.94	8.73	0.01
development + roads_barrier	-2076.58	10.08	0.01
landcover	-2071.76	14.91	0.00
global	-2066.67	20.00	0.00
linear (anthropogenic) facilitators	-2061.89	27.21	0.00
all_barriers	-2042.95	43.72	0.00
natural_barriers	-2041.78	44.89	0.00
null	-2002.73	83.94	0.00
***Full Population***			
global	-4034.82	0.00	1.00
all_barriers	-4018.70	16.12	0.00
natural_barriers	-4017.58	17.24	0.00
development + roads_barrier	-4015.69	21.45	0.00
natural_facilitators	-4012.56	22.27	0.00
landcover	-4010.79	24.03	0.00
all_facilitators	-3946.95	87.88	0.00
linear (anthropogenic) facilitators	-3662.09	118.48	0.00
null	-3537.85	496.97	0.00

^1^Forest, Forested Wetlands, Estuarine Wetlands, Water, Fields, Development, and Roads as barriers

^2^ all features

^3^ Forested Wetlands, Estuarine Wetlands, Water, Fields

^4^ Palustrine Emergent Wetlands, Scrub/Shrub Wetlands, Scrub/Shrub, and LiDAR

^5^ Palustrine Emergent Wetlands, Scrub/Shrub Wetlands, and Scrub/Shrub, Linear Facilitators (powerlines, railroads, roadsides), and LiDAR

^6^ Linear Facilitators: Railroads, powerlines, and roadsides

### Corridor identification

**Fig 2:**
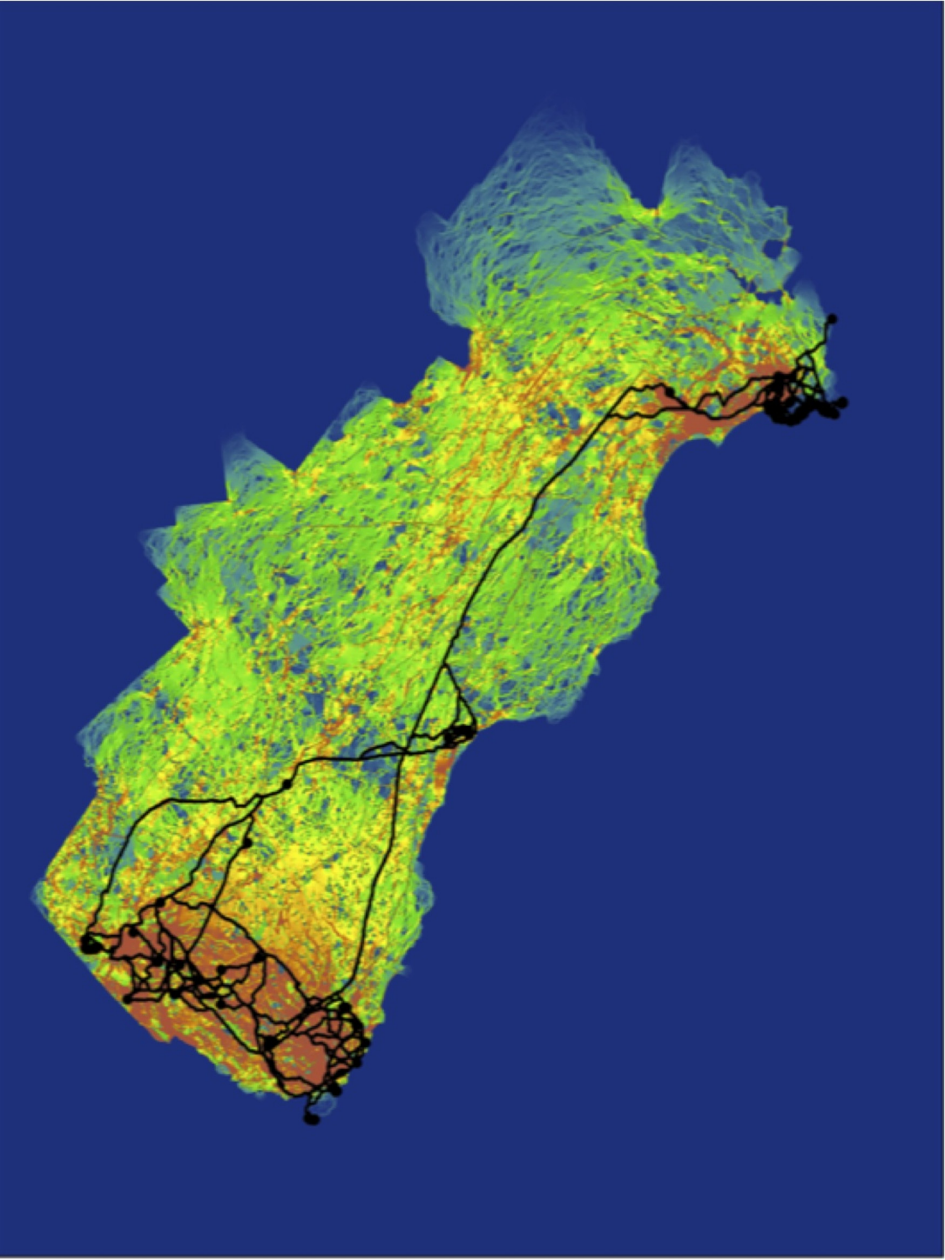
Connectivity corridors for cottontails. Circuit analysis overlayed with least cost analysis (black lines) of New England cottontail gene flow across the Maine-New Hampshire study area. Areas in red indicate high current flow/high probability of movement while green/blue areas indicate low probability of movement.

The Cape Elizabeth optimized resistance values were the best fit across the full population (highest partial Mantel *r*; S4 Table); this model included road classes 1–3 buffered with facilitating resistance values. These resistance values were used to evaluate the multivariate models for the full population prior to running corridor analyses. The full landscape model was the most explanatory mixed effect model across the entire population with clear model support (99% AIC weight; Table 4). Using the full landscape model, least cost path analysis identified a number of movement pathways within each population but only a limited number of routes connecting the two study areas (Fig 2). Similarly, current flow from circuit analyses was high within each population, although the individuals in Wells were isolated from Kittery by regions of lower current flow. Results of circuit analyses were nearly identical when considering individuals and focal areas; results from the focal area analysis are presented here. Between populations, least cost path identified minimal movement pathways and current flow from circuit analyses was relatively low outside of and between the occupied areas. In the southern portion of the study area, least cost path routes followed the railroad between western Kittery and Wells and the side of I-95 to the east, passing through regions of little or no current flow from circuit analysis. These two least cost path corridors converged to a single corridor north of Wells, following the roadside along I-95. In contrast, circuit analysis identified the coastal region as the most likely dispersal route between Kittery and Wells and highlighted a corridor from Kittery to Cape Elizabeth following a powerline right-of-way west of I-95.

## Discussion

### Functional Connectivity of Early Successional Obligates

Functional connectivity depends on the configuration and composition of the landscape [61]. Landscapes typically consist of a diversity of landcover types with varying degrees of permeability to animal movement [62]. Habitat types most suitable to movement are surrounded by a matrix of variably permeable landscape features. For specialists reliant on patchily distributed habitats, the composition and configuration of this matrix plays an important role in shaping dispersal patterns. Here we show that considering the full suite of matrix habitats is critical for understanding the dispersal of an early-successional-habitat specialist, the New England cottontail, for which suitable habitat patches account for less than 5% of the landscape.

Although it has been suggested that habitat suitability is a poor predictor of permeability to movement [7], the connectivity of New England cottontails appears to be driven by their preferred early successional habitat (scrub/shrub wetlands, scrub/shrub) as well as by anthropogenic features that include shrubby components (roadsides, powerlines, railroads). These latter habitat types likely provide sufficient cover and forage potential to act as stepping stone patches or dispersal conduits between occupied habitat patches. The distribution of these natural and human-modified shrubby habitats is critical for functional connectivity of cottontails, as the remaining landscape matrix features—including fields, forests, open water, and anthropogenic development—impede movement.

Development, fields, forest, and forested and estuarine wetlands account for approximately 90% of the coastal Maine and New Hampshire landscape. These landcover types are unsuitable for New England cottontail dispersal and other shrubland habitat specialists (e.g., [63–65]), likely due to their lack of dense vegetative cover. While LiDAR detected some shrub habitat within forest and forested wetland landcover pixels, the majority of forested areas in this study lack a dense understory required by early successional obligates. Areas of human development also lack suitable habitat and are typically correlated with roads, which also impede dispersal. Our results suggest that fields, which are used by many early successional species, including the congeneric eastern cottontails (*Sylvilagus floridanus*), are too open to provide functional dispersal pathways for New England cottontails. Early successional specialists with more generalist dispersal patterns [4,5] may be able to utilize fields as dispersal routes, while early successional obligates, such as the New England cottontail, may not.

### Roads as Both Barriers and Facilitators

Our results highlight the dual influence of roads as both barriers and facilitators of gene flow. Simultaneous positive-negative relationships with roads have been demonstrated in other studies, where connectivity is negatively influenced by road crossings but positively associated with movement parallel to roadways [66,67]. The barrier effects of roads are well established for a diversity of taxa, with varying life history strategies and habitat requirements [21,22]; the facilitating effects of roads are likely restricted to species that can take advantage of this habitat [17,68,69]. Recent landscape genetics investigations provide support for the hypothesis that the shrubby vegetation along roadsides has a positive influence on dispersal for some species [4,66]. By including roads as both facilitators and barriers within the same models, our findings support earlier work on the roadside hypothesis for New England cottontail [30,70]. Our approach, which gave high resistance to the pixels that comprised the roads and low resistance to a linear buffer alongside of the roads, improved the performance of multivariate models in predicting New England cottontail gene flow. The dual road influence was consistent across the local and population scales, suggesting that roads play an important and complex role in connectivity at both scales.

Our findings for the barrier effect of roads were consistent with a large body of research showing that high-traffic-volume roads pose barriers to gene flow for a diversity of organisms (reviewed in [22,68]) and have a greater influence on gene flow than secondary and unpaved roads [71,72]. In Kittery, I-95 and other major (classes 1–3) roads were found to have a greater resistance to movement than minor (classes 4–6) roads. In Cape Elizabeth, where no major highways occur, roads were not as influential in explaining cottontail gene flow when viewed as barriers, but were strongly influential as facilitators.

### Lidar—detected Habitats

We found that LiDAR imagery can be used to improve connectivity models [6] and identify dispersal corridors for early successional obligate species. Early successional habitats are difficult to characterize, especially when a shrubby understory occurs below a taller canopy structure. These habitats therefore may not be accurately represented by landcover data. To this end, LiDAR data, which describe plant canopy and subcanopy topographies [39], have proven useful in characterizing horizontal and vertical stand structure, including understory and ground cover [73]. LiDAR data have previously been shown to have value in ecological studies [74] and they have been used to improve habitat suitability models for managing wildlife species, including those with very specific vegetation requirements [75,76]. To our knowledge, this is among the first times LiDAR has been directly applied to connectivity analyses [77]. We used LiDAR to enhance our ability to identify early successional habitat and incorporated these LiDAR-detected habitats into our dispersal models. The LiDAR-identified scrub/shrub patches were a positive predictor of gene flow, despite covering only 1% of the landscape. The inclusion of LiDAR-identified habitat as a facilitator variable in multivariate models improved the correlation between effective and genetic distances over models that relied on Landsat-identified, scrub-shrub habitat alone. Our novel application of LiDAR demonstrates its utility in identifying dispersal habitat that may be difficult to identify using traditional landcover data, thereby aiding connectivity modeling, particularly for species reliant on distinctive vegetation structures.

### Comparison Across Landscapes

Identifying the consistencies and differences of genetic responses to landscape features as well as the factors that underpin dispersal patterns across a species' range can provide important insight for conservation management [78]. By replicating our study across two landscapes with different degrees of fragmentation, we were able to make inferences about features that consistently influence cottontail gene flow as well as how the influences on cottontail dispersal vary in relation to landscape context. While the two study areas had a similarly low proportion (<5%) of preferred early successional (scrub-shrub) habitat, they differed with respect to the matrix composition, including, amount of agriculture, levels of development, road density, average patch size, and cottontail densities. Based on these characteristics, we considered the Cape Elizabeth landscape less fragmented than the Kittery landscape. In comparing the results from these two landscapes, we find support for our hypothesis that gene flow of cottontails in the more fragmented Kittery landscape are more influenced by barrier features than by facilitating features, with the opposite pattern holding true for cottontails in the less fragmented Cape Elizabeth landscape.

Specifically, we found that forested wetlands, forest, and roads were influential barrier features in both landscapes, as well as across the entire study area as a whole–*i*.*e*., they had consistently elevated resistance values, relatively high Mantel correlations, and highest ranked mixed effect model fits. These features are therefore consistently influential for the species across a gradient of fragmentation. We also found that development, water, and fields had variable influences, depending on their degree of presence in the landscape: development was influential in the Cape Elizabeth landscape while water and fields were more influential in the Kittery landscape where they were more abundant (fields) or larger (Piscataqua River). Buffered roads and open water were the barrier models with the strongest explanatory power on gene flow in Kittery, consistent with earlier work in this system showing that roads and the Pisquataqua River subdivide genetically distinct groupings of cottontails in this region [30]. Models with natural facilitating features—scrub-shrub wetlands and scrub shrub—were more influential in Cape Elizabeth than Kittery. These features had relatively high support in both univariate and multivariate models—scrub-shrub wetlands was the top univariate model, while natural and all facilitators were the two highest ranked multivariate models. Comparatively, both univariate and multivariate facilitator models performed poorly (very low AIC model support) in Kittery, while models with barrier features had a stronger explanatory effect on gene flow. Interestingly, both natural (fields, forest, water, barrier wetlands) and anthropogenic (roads) barriers were important, likely given their prevalence in the Kittery landscape.

In addition to landscape context, considering spatial scale is also essential when making inferences from landscape genetics studies, as movement within the maximum dispersal distance of a species may vary dramatically from gene flow patterns across the entire population [56,57,79]. We considered local (within maximum dispersal distance—Kittery and Cape Elizabeth study areas) and regional (between populations) spatial scales. Our analyses revealed that several features, including roads, forest, and forested wetlands, act as strong barriers to New England cottontail gene flow at both the local and regional scales. Linear landscape features were consistently important as facilitators at both scales, although they were more influential at the regional scale where they provided critical corridors. Local analyses, however, identified differentially important landscape features within the two study areas; these important local influences would not have been identified by analyses at the regional scale. Accordingly, considering results from just one of the two local study areas would have provided an incomplete picture of landscape influences on cottontail gene flow. Extending implications of our findings across the species’ range, we suggest there are some consistent range-wide influences on gene flow with variation in locally important features, underscoring the need for habitat management priorities to be matched to the local landscape.

### Connectivity Corridors—Implications for Restoration

Corridors can provide critical linkages between habitat patches and wildlife populations [2] and can be identified by least cost path [80–82] or circuit [83,84] analysis. Comparative studies indicate that corridors identified by these two methods rarely overlap [85–87]. To identify potential corridors between the geographically isolated study sites of Kittery and Cape Elizabeth, we used both least cost path and circuit analyses across the entire study area and compared the results of the two approaches. We found that least cost path and circuit analyses identified similar connectivity patterns within each local study area, but differed substantially when considering long-distance dispersal pathways. Although the two methods identified different corridors between Kittery and Cape Elizabeth, they both identified pathways that followed linear strips of early successional habitat, including railroads, powerline rights-of-way, and roadsides, suggesting that these linear, anthropogenic habitats may function as important dispersal conduits for early successional specialists

Our results support previous conclusions about the relative strengths of least cost path and circuit analyses. Least cost path analysis identifies the single least costly path between a set of points [37], whereas circuit analysis considers all possible movement pathways, thereby accounting for flexibility in the movement behavior of multiple, individual animals and providing greater utility in planning management strategies and identifying locations for habitat restoration [83]. Consistent with expectations for landscapes with limited amounts of patchily distributed habitat [88], the high proportion of the landscape that is unsuitable for cottontail dispersal resulted in very few options for movement pathways. This pathway constraint was reflected more strongly in least cost path analyses, for which pathways converged to one or two options, while circuit analysis identified areas of high current flow outside of these least cost path routes. The powerline corridor identified by circuit analysis is a more biologically realistic pathway for long-distance cottontail dispersal than the interstate highway corridor identified by least cost path analysis. While both types of linear features enhance dispersal, the mortality risk associated with the interstate roadsides and the scarcity of stepping stone habitats in its vicinity makes it less desirable as a target for restoration activities than the powerline corridor. New England cottontails occupied habitat patches along this powerline corridor in the recent past [23,30], underscoring the importance of this area for focusing habitat restoration efforts to improve population connectivity in the future. To this end, circuit analyses also identified a large area of high current flow at the northern edge of this powerline, suggesting a clear strategy for focusing restoration.

## Conclusions

It has been hypothesized that species that specialize on patchily distributed habitats require a high ability to move through the landscape matrix to avoid the negative consequences of demographic isolation [89]. Accordingly, generalist dispersal patterns have been identified for several habitat specialists occupying naturally fragmented habitats [4,5,89]. In contrast, the New England cottontail, an early successional habitat obligate, is highly specialized on these sparse and patchy habitats in both its patch occupancy and dispersal. These naturally ephemeral habitats today exist in an extremely heterogeneous landscape matrix, fragmented by anthropogenic landscape modification. The majority of the matrix elements have a barrier effect on cottontail gene flow, particularly roads, development, forest, and forested wetlands, with the relative influence of these features dependent on the landscape context and composition. Only features comprised of natural or anthropogenic scrub-shrub habitats facilitate cottontail gene flow and these features comprise only a small percentage of the cottontail’s landscape. As a consequence, populations may become permanently isolated due to a scarcity of long-distance dispersal routes. Linear, anthropogenic, shrubby habitats, including powerline right-of-ways and roadsides, can serve as important corridors with potential for restoring connectivity. In fragmented landscapes, where shrubland habitat is limited, human-modified habitats may enhance functional connectivity by providing suitable dispersal conduits for early successional specialists. Restoration strategies that consider the value of these human-modified habitats may enhance conservation efforts to benefit a suite of vulnerable species dependent on endangered early successional ecosystems.

## Supporting Information

S1 Appendix Genetic distance matrices. Pairwise Rousset's *a* and *Dps* individual genetic distance matrices for New England cottontails in the Kittery and Cape Elizabeth study areas.
(XLSX)

S1 Table Optimal Resistance Values. Resistance values determined for each study area from best fit of New England cottontail genetic data with least cost path cost distances.
(DOCX)

S2 Table Mantel Results for Univariate Models. Mantel and partial Mantel r correlations of least cost path effective distances with New England cottontail gene flow for each landscape feature in Kittery and Cape Elizabeth study areas.
(DOCX)

S3 Table Mantel Test Resuls for Buffered Roads and LiDAR Models. Mantel and partial Mantel r correlations for models with the addition of buffered roads^1^ and LiDAR^2^ compared to models not including these features.
(DOCX)

S4 Table Results of Parameteration of Full Population Model Resistance Surfaces. Mantel and partial Mantel correlations between genetic distance and least cost path distances from models with alternate resistance values for the full study area extent.
(DOCX)
