# Supplementary material for: Anthropogenic Habitats Facilitate Dispersal of an Early Successional Obligate: Implications for Restoration of an Endangered Ecosystem
Source: PLoS One. 2016 Mar 8;11(3):e0148842. doi: 10.1371/journal.pone.0148842 (PMC4783018; doi:10.1371/journal.pone.0148842)
Supplement: S1 Table — Resistance values determined for each study area from best fit of New England cottontail genetic data with least cost path cost distances. (DOCX) [file pone.0148842.s002.docx]

**S1 Table.**

**Anthropogenic habitats facilitate dispersal of an early successional obligate: implications for restoration of an endangered ecosystem**

^1^Katrina E, Amaral, ^1,2^Michael Palace, ^3^Kathleen M. O’Brien, ^4^Lindsey E. Fenderson, ^1^*Adrienne I. Kovach

^1^University of New Hampshire, Department of Natural Resources and the Environment, 56 College Rd, Durham, NH 03824, USA; ^2^Institute for the Study of Earth, Oceans, and Space, Morse Hall, 8 College Road, Durham, NH 03824, USA; ^3^United States Fish and Wildlife Service, Rachel Carson National Wildlife Refuge, 321 Port Road, Wells, Maine 04090, USA;

^4^United States Fish and Wildlife Service, Northeast Fishery Center, Conservation Genetics Lab, P.O. Box 75, Lamar, PA 16848, USA; *Email: akovach@unh.edu

**S1 Table. Optimal Resistance Values.** Resistance values determined for each study area from best fit of New England cottontail genetic data with least cost path cost distances. "X" indicates the feature was not present within the analysis extent.

|  | **Feature** | **Kittery** | **Cape Elizabeth** |
| --- | --- | --- | --- |
| Development | Development (High) | 2 | 50 |
|  | Development (Med.) | 2 | 50 |
|  | Development (Low) | 2 | 50 |
|  | Development (Open) | 2 | 50 |
|  | Bare Land | 2 | 50 |
| Fields | Cultivated Crops | 10 | 2 |
|  | Pasture/Hay | 10 | 2 |
|  | Grassland | 10 | 2 |
| Forest | Forest (Deciduous) | 2 | 5 |
|  | Forest (Evergreen) | 2 | 5 |
|  | Forest (Mixed) | 2 | 5 |
| Wetlands | Palustrine Forested | 100 | 250 |
|  | Palustrine Scrub/Shrub | 1 | 1 |
|  | Palustrine Emergent | 1 | 1 |
|  | Estuarine Emergent | 5 | 2 |
| Water | Unconsolidated Shore | 10 | 2 |
|  | Open Water | 10 | 2 |
|  | Palustrine Aquatic | 10 | 2 |
|  | Estuarine Aquatic | 10 | 2 |
| Roads | Multi-lane highway (1) | 10 | NA |
|  | Primary Road (2) | 10 | NA |
|  | Secondary Road (3) | 10 | 50 |
|  | Improved (4) | 5 | 25 |
|  | Unimproved (5) | 5 | 25 |
|  | Trail (6) | 2 | NA |
| Scrub/Shrub | Scrub/Shrub | 1 | 1 |
