# Supplementary material for: Anthropogenic Habitats Facilitate Dispersal of an Early Successional Obligate: Implications for Restoration of an Endangered Ecosystem
Source: PLoS One. 2016 Mar 8;11(3):e0148842. doi: 10.1371/journal.pone.0148842 (PMC4783018; doi:10.1371/journal.pone.0148842)
Supplement: S2 Table — Mantel and partial Mantel r correlations of least cost path effective distances with New England cottontail gene flow for each landscape feature in Kittery and Cape Elizabeth study areas. (DOCX) [file pone.0148842.s003.docx]

**S2 Table.**

**Anthropogenic habitats facilitate dispersal of an early successional obligate: implications for restoration of an endangered ecosystem**

^1^Katrina E. Amaral, ^1,2^Michael Palace, ^3^Kathleen M. O’Brien, ^4^Lindsey E. Fenderson, ^1^*Adrienne I. Kovach

^1^University of New Hampshire, Department of Natural Resources and the Environment, 56 College Rd, Durham, NH 03824, USA; ^2^Institute for the Study of Earth, Oceans, and Space, Morse Hall, 8 College Road, Durham, NH 03824, USA; ^3^United States Fish and Wildlife Service, Rachel Carson National Wildlife Refuge, 321 Port Road, Wells, Maine 04090, USA;

^4^United States Fish and Wildlife Service, Northeast Fishery Center, Conservation Genetics Lab, P.O. Box 75, Lamar, PA 16848, USA

*Email: akovach@unh.edu

**S2 Table. Mantel Results for Univariate Models.** Mantel and partial Mantel r correlations of least cost path effective distances with New England cottontail gene flow for each landscape feature (optimized resistance value indicated in parentheses) in Kittery and Cape Elizabeth study areas, respectively. *P < 0.05; **P < 0.01; ***P < 0.001.

| **Kittery** | **Mantel *r*** | **Partial Mantel *r*** |
| --- | --- | --- |
| Forested Wetlands (100) | 0.392*** | 0.252*** |
| Estuarine Emergent Wetlands (5) | 0.387*** | 0.235** |
| Water (25) | 0.384*** | 0.233*** |
| Emergent Wetlands (Facilitator) | 0.380*** | 0.181** |
| Fields (10) | 0.376*** | 0.215** |
| Scrub/Shrub Wetland (Facilitator) | 0.376*** | 0.155* |
| Roads (Classes 1-6: 10,10,10,5,5,2) | 0.374*** | 0.227*** |
| Development (2) | 0.371*** | 0.196** |
| Forest (2) | 0.370*** | 0.159* |
| Scrub/Shrub (Facilitator) | 0.364*** | 0.122* |
| Linear Facilitators | 0.249*** | 0.170* |
| Isolation by Distance | 0.347*** |  |

| **Cape Elizabeth** | **Mantel *r*** | **Partial Mantel *r*** |
| --- | --- | --- |
| Forested Wetlands (250) | 0.180*** | 0.144** |
| Scrub/Shrub Wetland (Facilitator) | 0.156*** | 0.113** |
| Development (50) | 0.154*** | 0.111** |
| Forest (5) | 0.153*** | 0.108** |
| Estuarine Emergent Wetlands (2) | 0.148*** | 0.100* |
| Water (2) | 0.148*** | 0.100** |
| Fields (2) | 0.145*** | 0.096* |
| Scrub/Shrub (Facilitator) | 0.142*** | 0.091* |
| LiDAR | 0.140*** | 0.090* |
| Roads (Classes 1-6: x,x,50,25,25,x) | 0.139*** | 0.087* |
| Emergent Wetlands (Facilitator) | 0.136*** | 0.083* |
| Linear Facilitators | 0.123*** | 0.074* |
| Isolation by Distance | 0.109*** |  |
