# Supplementary material for: Anthropogenic Habitats Facilitate Dispersal of an Early Successional Obligate: Implications for Restoration of an Endangered Ecosystem
Source: PLoS One. 2016 Mar 8;11(3):e0148842. doi: 10.1371/journal.pone.0148842 (PMC4783018; doi:10.1371/journal.pone.0148842)
Supplement: S3 Table — Mantel and partial Mantel r correlations for models with the addition of buffered roads1 and LiDAR2 compared to models not including these features. (DOCX) [file pone.0148842.s004.docx]

**S3 Table.**

**Anthropogenic habitats facilitate dispersal of an early successional obligate: implications for restoration of an endangered ecosystem**

^1^Katrina E. Amaral, ^1,2^Michael Palace, ^3^Kathleen M. O’Brien, ^4^Lindsey E. Fenderson, ^1^*Adrienne I. Kovach

^1^University of New Hampshire, Department of Natural Resources and the Environment, 56 College Rd, Durham, NH 03824, USA; ^2^Institute for the Study of Earth, Oceans, and Space, Morse Hall, 8 College Road, Durham, NH 03824, USA; ^3^United States Fish and Wildlife Service, Rachel Carson National Wildlife Refuge, 321 Port Road, Wells, Maine 04090, USA;

^4^United States Fish and Wildlife Service, Northeast Fishery Center, Conservation Genetics Lab, P.O. Box 75, Lamar, PA 16848, USA

*Email: akovach@unh.edu

**S3 Table. Mantel Test Resuls for Buffered Roads and LiDAR Models.** Mantel and partial Mantel r correlations for models with the addition of buffered roads^1^ and LiDAR^2^, as compared to models not including these features. The addition of buffered roads or LiDAR improved model fit. These results signify that 1) roads exert a dual influence on cottontail gene flow, acting as barriers to perpendicular dispersal (road-crossing) while facilitating paralelle movement along shrubby roadsides; 2) LiDAR identifies additional shrubby habitat beyond what is identified using landcover data alon.

|  | Model | Mantel | p-value | Partial Mantel | p-value |
| --- | --- | --- | --- | --- | --- |
| Cape Elizabeth | All Facilitators | 0.1165 | 0.0126 | 0.0728 | 0.0885 |
|  | All Facilitators + LiDAR | 0.1231 | 0.0111 | 0.0826 | 0.0591 |
|  | Natural Facilitators | 0.1426 | 0.0029 | 0.0973 | 0.0265 |
|  | Natural Facilitators + LiDAR | 0.1483 | 0.0022 | 0.1053 | 0.0191 |
|  | Global model | 0.1821 | 0.0001 | 0.1482 | 0.0005 |
|  | Global model + roads 1-3 buffered | 0.2164 | 0.0001 | 0.1933 | 0.0001 |
|  | Global model + roads 1-6 buffered | 0.2217 | 0.0001 | 0.2002 | 0.0001 |
| Kittery | Global model | 0.3866 | 0.0001 | 0.2166 | 0.0004 |
|  | Global model + roads 1-3 buffered | 0.3971 | 0.0001 | 0.2584 | 0.0001 |
|  | Global model + roads 1-6 buffered | 0.3870 | 0.0001 | 0.2563 | 0.0001 |

^1^Road widths were given a barrier cost, with a 30-m pixel width buffered on either side given a low cost to model a facilitating effect on dispersal.

^2^LiDAR data were only available for the Cape Elizabeth study area.
