# Supplementary material for: Anthropogenic Habitats Facilitate Dispersal of an Early Successional Obligate: Implications for Restoration of an Endangered Ecosystem
Source: PLoS One. 2016 Mar 8;11(3):e0148842. doi: 10.1371/journal.pone.0148842 (PMC4783018; doi:10.1371/journal.pone.0148842)
Supplement: S4 Table — Mantel and partial Mantel correlations between genetic distance and least cost path distances from models with alternate resistance values for the full study area extent. (DOCX) [file pone.0148842.s005.docx]

**S4 Table.**

**Anthropogenic habitats facilitate dispersal of an early successional obligate: implications for restoration of an endangered ecosystem**

^1^Katrina E. Amaral, ^1,2^Michael Palace, ^3^Kathleen M. O’Brien, ^4^Lindsey E. Fenderson, ^1^*Adrienne I. Kovach

^1^University of New Hampshire, Department of Natural Resources and the Environment, 56 College Rd, Durham, NH 03824, USA; ^2^Institute for the Study of Earth, Oceans, and Space, Morse Hall, 8 College Road, Durham, NH 03824, USA; ^3^United States Fish and Wildlife Service, Rachel Carson National Wildlife Refuge, 321 Port Road, Wells, Maine 04090, USA;

^4^United States Fish and Wildlife Service, Northeast Fishery Center, Conservation Genetics Lab, P.O. Box 75, Lamar, PA 16848, USA

*Email: akovach@unh.edu

**S4 Table. Results of Parameteration of Full Population Model Resistance Surfaces.** Mantel and partial Mantel correlations between genetic distance and least cost path distances from models with alternate resistance values. The model that included resistance values from Cape Elizabeth univariate optimization and roads class 1-3 buffered as facilitators was the only model significantly correlated with gene flow when the effects of geographic distance were considered (partial Mantel r, p<0.05). The resistance values from this model were considered optimal for the full study area extent and were used to evaluate competing multivariate models for corridor analyses.

| **Multivariate Model** | **Mantel *r*** | **p-value** | **Partial Mantel *r*** | **p-value** |
| --- | --- | --- | --- | --- |
| **Expert Opinion Values** | 0.1813 | 0.0001 | 0.0255 | 0.2490 |
| **Optimized Cape E Values** | 0.1902 | 0.0001 | 0.0454 | 0.1120 |
| **Roads 1-3 buffered** | **0.2032** | **0.0001** | **0.0644** | **0.0408** |
| **Roads 1-6 buffered** | 0.1977 | 0.0001 | 0.0399 | 0.1426 |
| **Optimized Kittery Values** | 0.1919 | 0.0001 | 0.0431 | 0.1165 |
| **Average Optimized Values** | 0.1880 | 0.0001 | 0.0391 | 0.1530 |
| **Max Optimized Values** | 0.1887 | 0.0001 | 0.0425 | 0.1312 |
| **Isolation By Distance** | 0.1938 | 0.0001 |  |  |
